# Challenges of Type 2 Diabetes Mellitus Management From the Perspective of Patients: Conventional Content Analysis

**DOI:** 10.2196/41933

**Published:** 2022-10-27

**Authors:** Soghra Nikpour, Neda Mehrdad, Mahnaz Sanjari, Maryam Aalaa, Ramin Heshmat, Mahboobeh Khabaz Mafinejad, Bagher Larijani, Mahin Nomali, Tahereh Najafi Ghezeljeh

**Affiliations:** 1 Endocrinology and Metabolism Research Center Endocrinology and Metabolism Clinical Sciences Institute Tehran University of Medical Sciences Tehran Iran; 2 Elderly Health Research Center Endocrinology and Metabolism Population Sciences Institute Tehran University of Medical Sciences Tehran Iran; 3 Nursing and Midwifery Care Research Center Iran University of Medical Sciences Tehran Iran; 4 Osteoporosis Research Center Endocrinology and Metabolism Clinical Sciences Institute Tehran University of Medical Sciences Tehran Iran; 5 Department of e-Learning in Medical Education School of Medicine Tehran University of Medical Sciences Tehran Iran; 6 Chronic Diseases Research Center Endocrinology and Metabolism Population Sciences Institute Tehran University of Medical Sciences Tehran Iran; 7 Health Professions Education Research Center, Education Development Center Department of Medical Education Tehran University of Medical Sciences Tehran Iran; 8 Department of Epidemiology and Biostatistics School of Public Health Tehran University of Medical Sciences Tehran Iran

**Keywords:** diabetes mellitus, challenges, conventional content analysis, disease management, Iran

## Abstract

**Background:**

Patients with type 2 diabetes mellitus (T2DM) face significant challenges in the treatment process, which can have a negative impact on disease management. Proper management of the disease can reduce symptoms and complications, improve glycemic indices, and reduce mortality and readmission.

**Objective:**

Given the influential role of patients in prevention and self-care, this study was conducted to explore the challenges of diabetes management from the perspective of patients.

**Methods:**

Two rounds of focus group discussions with T2DM patients were conducted. The principal investigator of the study and a research assistant compiled a list of volunteer patients with names and contact information and selected participants based on their medical information. Participants were chosen via a purposive sampling technique. The questions were designed to encourage patients to share their views on how the treatment team communicates and participates in treatment, how they are trained, and the health care system. The discussion continued until data saturation. During 2 rounds of focus group discussions, the voices of the participants were recorded by 2 voice recorders, and one of the team members was a transcriber. After discussion, participant views were transcribed, and common issues were identified, sorted, and reported as categories and subcategories.

**Results:**

According to the conventional content analysis, 88 primary codes were extracted from the detailed and in-depth description of the participants. The codes were summarized after repeated readings and classified based on their similarities and semantic relevance. Through analysis and comparison, 4 categories and 7 subcategories were identified: communication challenges (poor medical staff communication, lack of psychological support), challenges to participation in treatment (lack of patient participation), educational challenges (training program bugs, inadequate training), and challenges of the health care system (inefficiency of the care system, caregiver inefficiency).

**Conclusions:**

This study showed that the treatment team members should pay more attention to the challenges of care and treatment from the perspective of patients with T2DM. Therefore, recommendations for future policies to overcome these obstacles include establishing a multidisciplinary health care team; using trained health care workers to provide organized treatment and care services; holding individual counseling sessions with patients in need of counseling; and providing counseling services, involving patients in the treatment and self-care process, and designing a comprehensive diabetes education program with an emphasis on education. Necessary information should be provided to the patients, and effective communicate should address patient concerns.

## Introduction

Type 2 diabetes mellitus (T2DM), with all its complications and consequences, constitutes the most critical problem for health systems worldwide. It is estimated that in 2014, 422 million adults lived with diabetes compared to 108 million in 1980 [1], which shows an almost 4-fold increase over a 34-year period. The prevalence of T2DM is expected to increase to 592 million adults worldwide in 2035 and 642 million adults by 2040 [2,3].

The prevalence of T2DM has affected all areas of the world, but the prevalence of T2DM is higher in low- and middle-income countries. It is estimated that about 12% of the total health expenditure in the world is spent on treatment of T2DM and related complications, and most countries spend 5% to 20% of their national health budgets on this disease [4]. In Iran, a national prevalence of 11.4% means that there are 4 million adults with diabetes. It is estimated that this number will reach 9.2 million by 2030 [5].

Management of diabetes is one of the most important priorities of the World Health Organization [6]. Diabetes complications threaten the health of patients and cause an economic burden on societies [7]. Studies have shown that for better management of diabetes complications and improvement of self-care, educational and counseling interventions should be provided by health workers [8,9]; however, patients’ knowledge and management of their disease are not always improved by educational interventions [10]. Evidence has shown that interventions based on lifestyle modification can be very efficient and effective in managing diabetes [11,12]. These interventions mostly rely on patient knowledge, attitude, behavior, and self-care to increase patient self-management, the strength of these interventions.

In other words, diabetes control requires a multidisciplinary care approach with a focus on self-management, in which the patient and family are at the center and the activity of all members of the treatment team is necessary to identify and cover the treatment and care needs of the patient and family [13]. Medical prescriptions and education are important dimensions of self-management, but behavioral and emotional dimensions are important as well. These dimensions are mainly focused on the patient and their emotions. Patients neglect their feelings and emotions when receiving the mass of information and education from health workers and cannot fully manage the condition. Patients must understand the disease, have a chance to adapt to the disease, and adjust the dimensions of their life with this disease. Multidisciplinary care can provide timely and effective care to patients [14]. Studies have shown that to achieve an effective prevention program and a comprehensive care program, understanding patients’ feelings and opinions about health conditions is essential. When adopting a comprehensive approach to diabetes care, we must consider patients’ feelings, concerns, fears, and expectations in our plans [15]. Because focus groups are used to explore perspectives on health issues, programs, interventions, and research [16], we used focus group semistructured discussions with groups of 4 to 12 people to examine a set of issues [17]. Managers usually start the discussion by asking broad questions about the topic of interest before asking focal questions. Although participants answer the facilitator’s questions individually, they are encouraged to talk and interact with each other [18]. This technique encourages respondents to explore and clarify individual and share perspectives [19].

One of the most important factors in controlling chronic disease is the participation of patients in disease management. Therefore, patients with diabetes and their families should learn about measures such as monitoring blood glucose, choosing an appropriate diet, and increasing physical activity [18]. Most of the studies have been conducted on the views of health workers regarding the disease and self-management of diabetes [17]. However, the patients who live with the disease must adapt to its symptoms and complications and manage the disease. Therefore, behaviors and feelings of patients are important dimensions of self-management and should be given prominence as an important indicator of the patient’s condition and compliance with treatment. Due to the pivotal and effective role of patients in the multidisciplinary team for the care and control of T2DM, the aim of this study was to investigate the challenges of T2DM management from the perspective of the patients.

## Methods

### Study Design

In this study, a qualitative methodology was selected and focus group discussion (FGD) was used as the study method. FGD is a qualitative research method in which the facilitator asks questions and participants volunteer their opinions, creating opportunities to extract information on a topic through group discussion. This also encourages participants to interact and exchange information about their experiences and perspectives [20-22]. Due to the interaction and effects of individuals on one another, FGDs show the dimensions of perception and knowledge of individuals, which are inaccessible through other methods of data collection [23]. A consolidated criteria for reporting qualitative research (COREQ) checklist is used to write the article [16].

### Participant Selection

Patients with T2DM were included in the study. Inclusion criteria were the ability to participate in the FGD without assistance (ie, understanding and speaking in Persian language and not having a cognitive impairment, which makes the interview difficult). Patients suffering from mental or physical disability were excluded from the study. After coordinating with the clinic supervisor, patients were simultaneously invited to participate in the FGD. Sampling was purposive. Two investigators (principal investigator and research assistant) selected study participants based on medical information. Background disease, disease duration, marital status, education level, type of drugs (oral antidiabetic drugs and insulin), and history of diabetes complications including retinopathy, nephropathy, diabetic foot ulcer, neuropathy, and cardiovascular diseases were recorded by the researchers. The researchers determined the time and place of the meetings in the clinic. The researchers had no previous relationship with any of the study participants, and they did not work at the clinic where this study was conducted.

### Setting

This study was performed at the Diabetes and Metabolic Diseases Clinic affiliated with Tehran University of Medical Sciences (Tehran, Iran). After providing written informed consent, participants were invited to participate in the FGDs. We arranged the patients in a circle position to improve group interactions and accurately record their voices. Each round of FGDs took less than 2 hours. At the beginning of each session, the principal investigator explained the objectives of the study to the participants and described their participation and activities in detail. The research assistant helped record the interviews, observe group interactions, take notes, and facilitate the discussion with probing questions.

### Data Collection

One researcher (ie, the principal investigator of the project) was as facilitator during the 2 rounds of FGDs, while another researcher took notes on the topics discussed and recorded participant reactions. Participants were asked to follow instructions explained to them at the beginning of each round of FGDs: each participant should introduce themself at the beginning, and they should not interrupt each other. Thus, the FGDs began with open-ended questions about challenges from the perspective of patients with T2DM.

The following questions were asked during the FGDs:

- What is your experience with how health care providers communicate?
- How do you feel about your participation in the management (both care and treatment) of your disease?
- What are your health care providers’ views on diabetes education?
- What are your main challenges in dealing with the health care system?

The facilitator encouraged the participants to express their opinions and experiences on related topics. The next questions were asked based on the answers of the participants. General promotional questions were asked to enrich the data: “What do you mean?” “Can you explain more?” “Can you give an example?” and “Would you like to mention anything else?”

The responses of the participants were recorded through 2 voice recorders and note-taking. We held 2 FGD rounds, with each round lasting less than 2 hours. First, 12 patients were selected. Two patients did not participate due to lack of time; 10 patients participated in the first round, and the same 10 patients participated in the second round. At the end of each FGD round, participants were asked to state what else they thought was important in the process of diabetes management and care.

### Data Analysis

The FGDs were ended when data saturation was reached. In other words, we continued the discussion until no new ideas were expressed or when the responses were similar or repetitive [24]. Conceptual transcription and mining were performed simultaneously. Conventional content analysis was done based on the 3 main steps of preparing, organizing, and reporting the analysis [25-27].

In the preparation phase, all recorded FGDs were transcribed. In case of any doubts on understanding the responses or any disagreement, the third researcher was consulted or a member check was applied. The transcribed FGDs were then read several times, and codes were identified. In the next step, similar open codes were classified into subcategories. Based on similarity and semantic relevance, subcategories were then divided into categories, and finally, categories, subcategories, open source, and key phrases were extracted from transcripts. In the final step, the analysis was reported.

### Trustworthiness

Trustworthiness is one of the most important dimensions of qualitative studies. According to Bowen [28], 4 reliable criteria including credibility, transferability, consistency, and conformability were considered in this study.

To ensure credibility, researchers tried to communicate well, spend enough time, and gain participants’ trust in data collection process. In order to do a member check, the results were returned to the participants to check the opinion of members and ensure the accuracy of the collected data. The research team also had a considerable conflict with qualitative data. In order to maintain transferability, we tried to avoid homogeneous participation by having the maximum variety in terms of age, sex, type of medication, disease duration, and history of diabetes complications, including retinopathy, nephropathy, neuropathy, cardiovascular diseases, and diabetic foot ulcer. To ensure consistency, all stages and research process were recorded and reported as thoroughly as possible.

### Ethics Approval

This study was approved by the research ethics committee of Tehran University of Medical Sciences in 2019 (approval: IR.TUMS.MEDICINE.rec.1397.847). All participants signed the written informed consent forms. Participants agreed to having their voices recorded, and the researchers made sure the data were anonymous and confidential at all times. The researchers promised that the information would remain confidential and the files and transcripts of interviews and voice records would be deleted at the end of the investigation.

## Results

### Participant Characteristics

Table 1 shows the characteristics of study participants. According to this table, 5 out of 10 participants were men aged 34 to 77 years, and 7 participants were married and lived in urban areas. Duration of the disease was between 10 and 27 years (Table 1).

According to the conventional content analysis, 88 codes were extracted from the rich and in-depth descriptions of the participants. The codes were summarized after repeated readings and classified on the basis of their similarities and semantic relevance. Through analysis and comparison, 4 categories and 7 subcategories were identified as shown in Table 2.

**Table 1:** Characteristics of the study participants.

No.	Gender	Age (years)	Marital status	Education level	Residential status	Type of medications	Disease duration (years)	Complications of diabetes
1	Man	50	Married	High school	Urban	Oral tablets and insulin	20	Cardiovascular disease
2	Woman	34	Single	High school	Urban	Oral tablets and insulin	10	Obesity Cardiovascular disease
3	Woman	60	Married	High school	Urban	Oral tablets and insulin	14	Retinopathy Hypertension Dyslipidemia
4	Woman	71	Married	High school	Urban	Oral tablets	26	Retinopathy Cardiovascular disease Skin dryness Skin itching
5	Man	53	Married	College	Urban	Oral tablets	14	Hyperthyroid
6	Man	77	Married	High school	Urban	Oral tablets	12	Retinopathy Nephropathy Cardiovascular disease Diabetic foot
7	Man	63	Married	College	Urban	Oral tablets	11	Retinopathy Hypertension Dyslipidemia
8	Woman	74	Widow	High school	Rural	Oral tablets	22	Retinopathy Cardiovascular disease Hypertension
9	Woman	61	Widow	High school	Rural	Oral tablets and insulin	27	Retinopathy Hypertension Dyslipidemia Digestive problems
10	Man	74	Married	Illiterate	Rural	Oral tablets and insulin	17	Retinopathy Cardiovascular disease Hypertension Dyslipidemia Anorexia Gastrointestinal upset

**Table 2:** Categories, subcategories, and codes discovered in the study.

Category/Subcategory		Code
**Communication challenges**		
	Poor medical staff communication	Insufficient understanding and empathy Unkindness and disrespect No face-to-face communication Inflexibility of health staff
	Lack of psychological support	Creating fear in the patient about signs and consequences of the disease Change of doctor due to inappropriate behavior of the doctor Lack of individual counseling Lack of suitable space for consultation and haste of health staff
**Challenges to participation in treatment**		
	Lack of patient participation	Not giving the patient the right to comment Lack of explanation about the treatment process: complications, alternative therapies and duration of treatment The patient is not involved in nutrition
**Educational challenges**		
	Training program bugs	Continuity of training programs Training programs do not start on time Advertising for fellow physicians in training programs
	Inadequate training	Dissatisfaction with the educational information provided and its duration Get additional information from other educational resources Providing information in an authoritarian manner Lack of adequate training in the diagnosis and treatment and complications of the disease Not giving enough training in self-care Not teaching about traditional medicine
**Challenges of the health care system**		
	Inefficiency of the care system	Short visit by a doctor Not seeing a doctor even by paying for a visit Lack of access to the relevant specialist (in person or by phone) Do not do specialized patient work as a team Duplicate record making for the patient
	Caregiver inefficiency	Do not follow the patient’s treatment

### Communication Challenges

Poor communication by medical staff included insufficient understanding and empathy, unkindness and disrespect, lack of face-to-face communication, and inflexibility of health workers. Patients expressed their opinions that health personnel do not care about patients and the disease, and members of the diabetes treatment team did not have a close relationship with and did not listen to patients.

Disrespect by the medical staff was expressed from the patient’s point of view.

The patient who cried while talking and said I live alone and I’m nervous. I would like them to talk to me and support me.

Participant 6

Patients wanted the doctor to pay attention and make eye contact when talking to them.

The doctor is also working on his computer while talking to me and this behavior bothers me.

Participant 5

Lack of psychological support was another subcategory. Patients were worried about their symptoms and illness, and the health care staff did not address their complaints or spend enough time to listen to them (see examples in Textbox 1). Patients sometimes changed doctors because of this.

Communication problems between patient and health care provider are caused by various factors. The patient’s condition and perspective help to determine these factors. Patients with diabetes have many different meetings with health care providers due to the symptoms and control of the disease, and the issue of communication is of great importance for these patients. On the other hand, many doctors and nurses do not answer the questions of these patients because they believe that they received enough training.

Therefore, communication problems can be an obstacle in the process of patient management and cause noncompliance with treatment. It is necessary to solve this problem by considering the patient and the patient’s emotions and feelings.

**Illustrative quotations about lack of psychological support.**

- My blood sugar drops late at night and I don’t know what to eat. [Participant 3]
- When I eat something, my blood sugar rises to 300 at once and I get very worried. When I tell my problem, they tell me to eat less. [Participant 6]
- When I have hypoglycemia, I feel very drowsy. I do not have the patience to talk to anyone, I feel very hot, and it’s hard to breathe so I open the windows. [Participant 4]
- I have severe dry mouth, this symptom annoys me a lot. [Participant 1]
- Lack of appetite control and overeating. Sometimes I overeat and I like to eat everything and I don’t pay attention to what people around me say. [Participant 2]
- I do not feel like going to exercise, even though I know I should be physically active. [Participant 8]
- I get full early when I eat. [Participant 7]
- I get very nervous when my blood sugar rises. [Participant 9]
- Sometimes my toe hurts and sometimes at night my feet get very cold and my toes get cold and hot. [Participant 5]
- I worked for a food company. I had severe itching and bleeding from scratching, and I did not know what to do. I went to the doctor. He asked about my workplace and then said “You are miserable because your blood sugar is above 400. Do not eat sweets at all.” I was very scared. Good behavior of the doctor in the office with the patient is very important. A patient complained about the doctor’s bad behavior and had to change his doctor. [Participant 10]
- I had to change my doctor because of uncaring behavior. He didn’t answer my questions and got angry and threw me out of his office once. [Participant 8]

### Challenges to Participation in Treatment

Challenges to participation in treatment has one subcategory, lack of patient participation, which originates from not giving the patient the right to comment; lack of explanation about the treatment process including complications, alternative therapies, and duration of treatment; and not involving the patients in nutrition programs and physical activity.

Patients like to participate in the treatment and care process, including the type of medications, nutrition, and exercise. They also like the health care providers to explain why these things should be done for them. They stated that decisions are often made for them without considering the their opinion.

The doctor did not allow me to comment on my illness and kicked me out of the room.

Participant 3

Nonparticipation in treatment, changing medications, and lack of a diet program were challenges some patients encountered.

The medical staff made a quick decision about how to treat my illness. At first, I only took pills for diabetes. When my A1C was close to 10, they told me to take my insulin first, and they did not give me any training or consultation.

Participant 8

They did not involve me in how to eat with diabetes; they just gave me a plan and told me to do it.

Participant 9

I have a lot of appetite and I am not satisfied with the diet that the health staff has prepared for me without my consultation and participation.

Participant 1

Not paying attention or listening and neglecting the patient creates a mental burden for the patient. The patient feels that the doctor repeats the medication orders in a routine and usual manner. Involving the patient in the treatment process can strengthen the trust and relationship between patient and treatment team.

### Educational Challenges

Participants cited educational challenges in the form of training program bugs, such as continuity of training programs, not starting training programs on time, and advertising for fellow physicians in training programs. In addition, patients stated that training programs should be continued and sometimes training programs are not held. Training programs usually do not start on time. In training programs, doctors advertise to their colleagues and this is not acceptable.

Training programs do not start on time and are advertised for another doctor.

Participant 6

Training programs are not held regularly.

Participant 9

Complaints of inadequate training consisted of dissatisfaction with the educational information provided and its duration; difficulty getting additional information from other educational resources; and inadequate training in the diagnosis, treatment, and complications of the disease and about self-care. Additional complaints were raised about not teaching traditional medicine and providing information in an authoritarian manner. The patients complained that they were not given enough and correct information about the time to take pills in the educational programs. They also liked to be given information about the use of herbal medicines.

They do not teach me about taking drugs. I took diuretic pills at night. I had frequent urination. Today, when I had a problem, I was taught that I should take this pill in the morning. When the training here is not complete, we have to use virtual space to find answers to our questions, which we do not know are valid.

Participant 10

Patients also complained of insufficient training and incomplete education about self-care.

A few years ago, when my blood sugar was high, they told me that I had diabetes and they gave me 300 ml of vitamin B1, and they told me to walk more, and they did not teach me about diet and disease.

Participant 7

The first time I had high blood sugar and they said I had diabetes, my blood sugar was 180 and they gave me half a pill of Goli Bin Kalamid and they did not tell me what to eat and what not to eat and they just told me not to eat sweets.

Participant 9

When I was diagnosed with diabetes, I was only taught to walk and I was not taught about nutrition.

Participant 8

They do not teach about traditional medicines.

Participant 1

They do not answer me when I talk about using herbs to lower blood sugar.

Participant 2

### Challenges of the Health Care System

Inefficiency of caregivers and the care system were identified as challenges in the health care system. According to the patients, insufficiency of the care system included short visits with the doctor, not seeing a doctor even if paying for a visit, lack of access to relevant specialists (in person or by phone), lack of provider team work, and duplicate record making for the patient.

The duration of the doctor’s visit is very short. The doctor’s visit time is very short. The doctor quickly examines and prescribes medicine, and this makes me very uncomfortable in every visit. Whenever I protest, he says that he should visit other patients as well.

Participant 1

Patients also complained that sometimes they paid for a doctor’s visit but were not visited by a doctor.

Although we pay for a doctor’s visit, and sometimes we even pay for two doctor visits, but we do not see the doctor, and then the survey form is texted that you are satisfied with the doctor? And they expect me to fill in the evaluation form.

Participant 5

In addition, the patients were upset that things were not done by the team in an organized manner, and they had to visit several times to get services.

Things are not done as a team. I have to visit a doctor once and another day for nutrition counseling.

Participant 2

Patients were dissatisfied with the medical record filing system, and the system was not working well. Each new group that comes to the clinic creates a new file for the patient.

I have a record in this diabetes clinic for 18-17 years. The new people who came filed a record for me again.

Participant 4

Another challenge was caregiver insufficiency. The most important issue raised by patients regarding caregiver efficiency was follow-up.

The first time my blood sugar was high, the doctor told me to get rid of stress and adjust my diet and walk for an hour a day. The next time I went, he said my blood sugar was low and I did not take any further action because the doctor did not insist on taking medication. He did not follow up and the next time I went; my blood sugar was so high that I had to inject insulin.

Participant 9

## Discussion

### Principal Findings

Due to the important and effective role of patients in better prevention and self-care, focus group meetings were held to review the challenges of patients with diabetes for disease management. The results showed their challenges in 4 main categories including communication challenges, challenges to participation in treatment, educational challenges, and challenges of the health care system.

Communication problems in the treatment staff and training program indicate ineffective communication and inadequate patient education. The results of a study investigated the barriers to diabetes care in a developing country. This study showed that patients need more time to understand the content of the educational program provided by the treatment team, and the treatment team needs to allocate more time to patients [29]. Another study found that communication discordance with the health care members was an obstacle to understanding diabetes education [30]. Another study determined that communication skills of health system employees are very important in the management of patients with diabetes, and they may be able to have a greater impact on the patient’s perception through effective communication skills [31]. A third study reported that according to the patients’ experiences of communication with health care providers, factors such as trust and confidence, willingness to communicate, attention to the patient’s emotional dimension, and the appropriateness of the meeting time and conditions are essential in effective communication [32]. Therefore, it seems that communication is a key for achieving better self-management in patients. In several previous studies, unprofessional behavior of the medical staff with patients was not directly mentioned, but in this study, patients complained of insufficient understanding and empathy, unkindness and disrespect, and lack of face-to-face communication and flexibility of the medical staff, all of which are classified as communication challenges [33-36].

One of the most important factors in controlling this disease is the participation of patients in the treatment [37]. The results of the study showed that in order to achieve an effective prevention program and comprehensive care program, the presence, feelings, and understanding of patients about their health condition are necessary [15]. Our study highlighted that one of the challenges in the management of the patients with T2DM was lack of patient participation. Review of the literature also showed that patient activation and patient involvement in treatment play a crucial role in self-management of patients with T2DM [38-40]. Recent review article determined that patient activation can be used as a reliable tool for improving T2DM self-management and clinical outcomes [38]. Another study was found a relationship between patient empowerment, self-management education, and lifestyle modification in the management of patients with diabetes [39]. Patient empowerment can cause more coordination between the treatment staff and the patients. In addition, the awareness of the patients about their condition and disease increased by empowering them.

Our study showed that patients face educational challenges in the management of diabetes. In a study on barriers to self-care, diabetics requested more time for visits and counseling. They also needed to continue their education in different ways and update their information on diabetes care [41]. Another similar study on barriers to self-care education for diabetics from the perspective of nurses and patients showed that one of the main reasons for inadequate self-management in patients with diabetes is the existence of barriers in educating these clients [42]. A study in Bangladesh found that to better understand patients’ views on diabetes and drug beliefs and identify psychological stress, health care providers should provide quality health education interventions and more up-to-date information to patients [43]. A study in Pakistan on patients’ perspectives, experiences, and barriers toward diabetes-related self-care reported that counseling by health care providers is the key enabler that encourages study participants to adhere to diabetes-related self-care practices [44]. Lack of understanding about diabetes medication management and long-term safety of diabetes medications could be the examples of poor inadequate training [30].

The results of this study showed that obstacles in the management of diabetes from the patients’ view are problems related to the care system and health system. Lack of support for patients was one of the most important problems mentioned in previous studies [31,43,45]. Financial and social support can effectively help to better manage the disease [46]. The lack of support for patient caregivers was also an obstacle to disease management from the patients’ view [47]. The results of the study showed that patients need to talk to the treatment team about emotions, such as anxiety, frustration, and inattention, and need their support. This may be more the case in developing countries where there is a shortage of specialist clinics and time constraints on multidisciplinary diabetes treatment teams. Studies also require group consultation with patients and experiences. One of the main reasons for inadequate self-management in diabetics is the existence of barriers in educating these clients. The care system provides insufficient support for patients in the field of medications and therapeutic interventions. Diabetes is a chronic disease requiring medication regimens and regular visits to providers. Therefore, support measures such as insurance coverage and reduction the number of drugs and treatment costs should be considered for these patients [48].

### Limitations

The most important limitation of this study was related to the nature of the study methodology. The generalizability of the qualitative study is limited. Another limitation was that only 2 rounds of FGDs were held, fewer than the researcher expected. In qualitative studies, data saturation is a definite determinant of ending the study. In this study, due to the similarity in patients’ opinions and the lack of a new opinions and ideas after 2 rounds of FGDs, this factor may not affect the findings.

### Conclusions

The results showed that patients pay more attention to nontherapeutic issues than therapeutic issues. Communication with the patient, patient education, proper support from the health system, and adequate participation in treatment were challenges that made the process of treatment and self-management difficult for patients. Therefore, it is necessary to pay attention and check these cases in the management of the patient. A comprehensive training program should be designed to address these patient concerns.

## Abbreviations

COREQ: consolidated criteria for reporting qualitative research
FGD: focus group discussion
T2DM: type 2 diabetes mellitus
